# *Tolypothrix* Strains (Cyanobacteria) as a Source of Bioactive Compounds with Anticancer, Antioxidant and Anti-Inflammatory Activity

**DOI:** 10.3390/ijms26115086

**Published:** 2025-05-26

**Authors:** Ivanka Teneva, Tsvetelina Batsalova, Dzhemal Moten, Zhana Petkova, Olga Teneva, Maria Angelova-Romova, Ginka Antova, Balik Dzhambazov

**Affiliations:** 1 Department of Botany and Biological Education, Faculty of Biology, Paisii Hilendarski University of Plovdiv, 24 Tsar Assen Str., 4000 Plovdiv, Bulgaria; teneva@uni-plovdiv.bg; 2 Department of Developmental Biology, Faculty of Biology, Paisii Hilendarski University of Plovdiv, 24 Tsar Assen Str., 4000 Plovdiv, Bulgaria; tsvetelina@uni-plovdiv.bg (T.B.); moten@uni-plovdiv.bg (D.M.); 3 Department of Chemical Technology, Faculty of Chemistry, Paisii Hilendarski University of Plovdiv, 24 Tsar Assen Str., 4000 Plovdiv, Bulgaria; zhanapetkova@uni-plovdiv.bg (Z.P.); olga@uni-plovdiv.bg (O.T.); maioan@uni-plovdiv.bg (M.A.-R.); ginant@uni-plovdiv.bg (G.A.)

**Keywords:** *Tolypothrix*, cyanobacteria, fatty acids, cytotoxicity, anticancer activity, antioxidant activity, anti-inflammatory activity

## Abstract

Cyanobacterial extracts offer significant potential for the development of new natural antioxidants and biologically active compounds with applications in various industries. Data on the genus *Tolypothrix* are limited; therefore, the aim of the present study was to investigate the anticancer, antioxidant and anti-inflammatory activity of extracts prepared from strains of this genus. Cytotoxicity and anticancer activity were evaluated by in vitro tests with four cell lines using the MTT assay. The assessment of antioxidant activity was performed by the DPPH and ABTS methods in combination with the calculation of the total phenolic content. Anti-inflammatory activity was investigated using the LPS-stimulated macrophage model (RAW264.7) and subsequent measurement of the levels of secreted cytokines IL-6 and TNF-α. The lipid content and fatty acid composition of the non-polar extracts were determined by gas chromatography (GC). To elucidate the mechanism of cytotoxicity/anticancer action of the non-polar extracts, the effects of stearidonic acid, which was detected in four of the studied cyanobacterial strains, were additionally tested on the same cell lines. A molecular docking analysis was performed simulating the interaction between stearidonic acid and its target molecules and receptors (ALOX5, COX-2, NF-kB and PPAR-γ). In all cancer cell lines (but not in the normal one), dose-dependent cytotoxic effects were observed after exposure to different concentrations of non-polar *Tolypothrix* extracts. The most pronounced inhibitory effect was observed on the HT-29 cell line, with an IC_50_ value of 106.27 µg/mL. A dose-dependent antioxidant effect was established for all tested extracts, measured by both DPPH and ABTS methods. All non-polar extracts reduced the production of pro-inflammatory cytokines IL-6 and TNF-α in LPS-stimulated macrophages RAW264.7, and the effects were dose-dependent. Analysis of the fatty acid composition revealed 26 different fatty acids. Our conclusion is that the *Tolypothrix* strains exhibit anticancer, antioxidant, and anti-inflammatory activity and could be a promising source for the production of natural products.

## 1. Introduction

Cyanobacteria are considered one of the most promising groups of organisms, producers of biologically active natural products, whose composition and potential, however, are still not fully understood. Cyanobacteria produce an extremely diverse range of peptide antibiotics, lipopeptides, amino acids, fatty acids, macrolides, esters, indoles, alkaloids, amides, lactones, polysaccharides and other biologically active substances with enormous potential as drugs and biocontrol agents [1,2,3].

Cyanobacterial metabolites have been found to exhibit various biological activities—tumor suppressor, tumor promoter, immunomodulatory, anti-inflammatory, antifungal, antibacterial, antiviral, antiprotozoal and anthelmintic [4,5,6,7,8,9,10]. However, the mechanisms by which a large number of cyanobacterial natural products exert their effects are not fully understood. Therefore, studies in this direction are relevant and important. Currently, only about 10–20% of cyanobacterial secondary metabolites have been characterized in terms of their chemical structure and biological potential [11,12]. On the other hand, the variability of the chemical structures produced by cyanobacteria enables interaction with different cellular targets, leading to hypotheses for new mechanisms of cyanobacterial action [13,14].

Many species of cyanobacteria produce cyanotoxins as secondary metabolites. These toxins and the species that produce them are the subject of intense research because of the danger they pose to human health and the potential ecological risk during “blooms”. On the other hand, cyanotoxins can also be beneficial. Cyanobacterial neurotoxins, for example, and in particular anatoxin-a(s), have antiphosphatase and anticholinesterase effects [15,16,17,18]. Antiacetylcholinesterase action of any compound may play an important role in the search for potential drugs against Alzheimer’s disease (AD), where the symptomatic treatment is restoration of cholinergic function by inhibition of acetylcholinesterase. Thus, anatoxin-a(s) may be useful in such neurodegenerative conditions [18,19]. Therefore, knowing the producers of cyanotoxins as well as the types of cyanotoxins they produce is important.

Cyanobacteria have a significant potential for the production of fatty acids (lipids), some of which are essential. These fatty acids are involved in important metabolic pathways, determining the crucial role of their biological activity for the human organism [20]. Fatty acids can be taken as dietary supplements and thus improve our lifestyle and quality of life. In addition, they can and are used as biofuel feedstock [21].

The cyanobacterial genus *Tolypothrix* is poorly studied in terms of its production of biologically active substances. Some *Tolypothrix* species produce unique polysaccharides (exopolysaccharides) that may have anti-inflammatory, immunomodulatory or antioxidant properties [22]. *Tolypothrix tennuis* has been reported as a producer of exopolymers consisting mainly of carbohydrates (17–50%), protein (4.4–7.2%), uronic acid (4.7–9.5%) and sulfate (0.6–6.6%) that could stop bleeding or be used as blood clotting agents for wound healing [22].

Other isolates of *Tolypothrix* produce substances with antibiotic and/or antimicrobial properties, making them the subject of research for development of new drugs. An example of such a substance is 2, 4-bis (2-methyl-2-propanyl)phenol-phosphorous acid isolated from *Tolypothrix rechingeri* [23].

Members of the genus *Tolypothrix* have been reported as producers of hassallidins [24]. Hassallidins have demonstrated antifungal activity against opportunistic pathogenic fungal species [24]. Other biologically active substances with antifungal activity, isolated from *Tolypothrix tjipanasensis* and *Topypothrix byssoidea*, respectively, are glycosides named tjipanazoles and cyclic tridecapeptides (tolybyssidins A and B) [25,26].

The production of antitumor compounds by members of the genus *Tolypothrix* is also being investigated, with some of these compounds showing promising results in preclinical trials. Tolyporphins isolated from *Tolypothrix nodosa*, for example, have been shown to photosensitize tumor tissue [27].

The antiproliferative activity of metabolites released from *Tolypothrix* sp. was tested using A549 (human lung cancer) and HEK-293 (human normal kidney) cell lines. The compounds showed significant cytotoxic activity against the cancer cell line A549, while on normal human cells, HEK-293, they had almost no effect [28]. According to the authors, the antitumor effect may be due to the 2,6,10-trimethyl, 3,7,11,15-tetramethyl-2-hexadecen-1-ol, hexadecanoicacid, heneicosanoic acid and hexadecanoate found in the extract. A summary of the pro- and anti-carcinogenic effects of fatty acids is provided by Westheim et al. [29]. Overall data show that, as a rule, saturated fatty acids (SFAs), such as palmitic acid, promote tumor progression. The effect of monounsaturated fatty acids (MUFAs) such as oleic acid is unclear. Some omega-6 polyunsaturated fatty acids (PUFA), such as arachidonic acid, have pro-carcinogenic effects, while omega-3 polyunsaturated fatty acids (α-linolenic, eicosapentaenoic, and docosahexaenoic) generally have anti-carcinogenic effects [29].

Representatives of the genus *Tolypothrix* produce compounds with anti-inflammatory properties, which makes them interesting for the development of new medicines for the treatment of inflammatory diseases. An example of such a substance is tolypodiol isolated from *Tolypothrix nodosa* [30].

The generally accepted strategy for evaluating the potential of cyanobacteria as producers of biologically active substances involves the study of “crude” extracts and fractions. In vitro and/or in vivo test systems are used to measure antitumor and antioxidant activity, and the levels of pro-inflammatory cytokines such as TNF-α, IL-1β and IL-6. Anti-inflammatory effects were reported for two digalactosyldiacylglycerols (DGDGs) isolated from *Nodularia harveyana* [31]. The authors found decreased levels of TNF-α and NF-κB expression in LPS-stimulated THP-1 (human leukemia monocytic) cells after treatment with these two digalactosyldiacylglycerols [31]. Low levels of TNF-α, IL-1β and IL-6 were reported in LPS-stimulated RAW264.7 macrophages and mouse bone marrow-derived macrophages after treatment with lipid fractions from *Nostoc commune* var. *sphaeroides* and *Spirulina platensis* [32].

The study of the biologically active substances isolated from representatives of the genus *Tolypothrix* continues, and the potential for the discovery of new and significant compounds with diverse applications is high. The aim of the present study was to investigate the anticancer, antioxidant and anti-inflammatory activity of extracts from five selected cyanobacterial strains of the genus *Tolypothrix* and to analyze the chemical composition of the potential bioactive substances. An attempt has been made to determine and discuss the mechanisms by which these bioactive substances exert their specific effects.

## 2. Results

### 2.1. Cytotoxicity and Anticancer Potential of Tolypothrix Extracts

**Figure 1:**
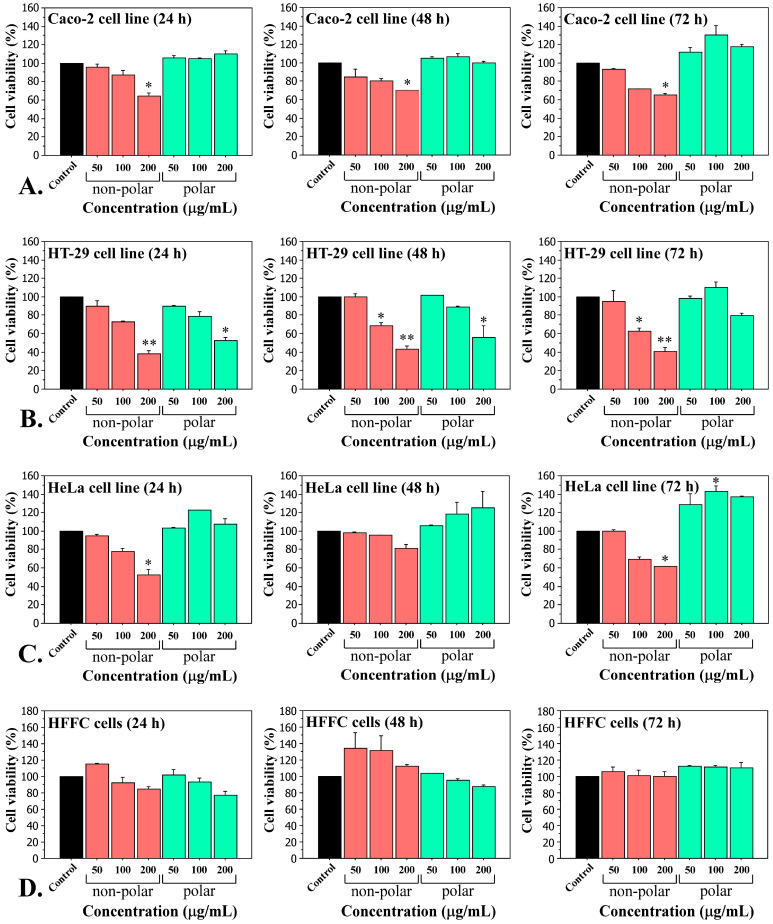
Cytotoxicity of *Tolypothrix tenuis* PACC 5497 extracts on adenocarcinoma Caco-2 cells (**A**), HT-29 cells (**B**), HeLa cells (**C**) and normal human fibroblasts HFFC (**D**). Cell viability was measured using MTT assays after treatment with increasing concentrations of extracts (50, 100, and 200 µg/mL) for 24, 48 and 72 h. Results are expressed as mean ± SD of three independent experiments, each performed in triplicate. Statistical significance was defined by the Mann–Whitney U test versus the control. * *p* < 0.05, ** *p* < 0.01.

The non-polar and polar cyanobacterial extracts from five *Tolypothrix* strains were used in experiments on human carcinoma cell lines (Caco-2, HT-29, HeLa) and normal fibroblasts (HFFC). The cytotoxic effects and anticancer potential were determined using the MTT assay. Dose-dependent cytotoxic effects were observed in all cancer cell lines following the exposure for 24, 48 and 72 h with various concentrations (50, 100 and 200 µg/mL) of non-polar *Tolypothrix tenuis* PACC 5497 extract, compared to the control (Figure 1A–C). These data showed that the most pronounced inhibition was observed on the HT-29 cell line, with an IC_50_ value of 106.27 μg/mL (Figure 1B). In contrast, the polar extract of *Tolypothrix tenuis* PACC 5497, in most cases, does not alter the viability of carcinoma cell lines and even stimulated their vital activity and proliferation (Figure 1). Both polar and non-polar extracts of *Tolypothrix tenuis* PACC 5497 did not significantly affect the viability of normal human fibroblasts (HFFC) (Figure 1D).

Similar results have been observed when treating cells with extracts from the other strains studied (*T. tenuis* PACC 8648, *T. distorta* CCALA 194, *T. distorta* SAG 1482-2, and *Tolypothrix* sp. PACC 5501) (Figures S1–S4, Supplementary Materials). Based on the results obtained from the in vitro cytotoxicity tests, we decided to continue our studies with only non-polar extracts, as they were found to have cytotoxic effects on carcinoma cell lines, but not polar extracts.

### 2.2. Lipid Composition of Tolypothrix Extracts

The content of glyceride oil of the five *Tolypothrix* samples was determined. The results are given in Table 1.

All analyzed strains were characterized with very low oil content, lower than 1.0%. The oil content of *Tolypothrix distorta* CCALA 194 and *Tolypothrix distorta* SAG 1482-2 was found to be similar (0.31 and 0.45%, respectively), as well as the oil content of *Tolypothrix tenuis* PACC 8648 and *Tolypothrix tenuis* PACC 5497 (0.97 and 0.72%), respectively. An exception was the strain *Tolypothrix* sp. PACC 5501, in which it was 1.09%. The oil content of all analyzed samples was close to that of other plants with low amounts of glyceride oil, such as for the moss *Hypnum cupressiforme* (1.27%), reported earlier [33].

The fatty acid composition of the glyceride oil of five cyanobacteria species was established. The results are presented in Table 2.

The fatty acid composition analysis revealed 26 different fatty acids, with the highest levels being palmitic (C16:0), oleic (C18:1), and linoleic (18:2) acids across all oils. The sample *Tolypothrix* sp. PACC 5501 stands out with 36.2% palmitic acid, followed by 35.2% in *Tolypothrix tenuis* PACC 8648 and 32.0% in *Tolypothrix tenuis* PACC 5497. The rest of the analyzed samples, *Tolypothrix distorta* CCALA 194 and *Tolypothrix disorta* SAG 1482-2, contained 21.3% and 24.9% palmitic acid, respectively. Our results significantly differ from those reported earlier [34], which noted 17.5% in *Tolypothrix* sp. Prague and 16.1% in *Tolypothrix* sp. Tatra M. Oleic acid was the second most predominant fatty acid, ranging from 10.2% to 21.8% in all five analyzed samples. *Tolypothrix distorta* CCALA 194, *Tolypothrix distorta* SAG 1482-2, and *Tolypothrix tenuis* PACC 5497 had comparable quantities of this acid, while *Tolypothrix* sp. PACC 5501 and *Tolypothrix tenuis* PACC 8648 had nearly half as much. High levels of palmitoleic acid were found in *Tolypothrix distorta* CCALA 194 and *Tolypothrix disorta* SAG 1482-2 (13.5% and 15.9%, respectively), with the lowest amount in *Tolypothrix tenuis* PACC 5497 (2.3%). Other reports indicated palmitoleic acid levels of 11.2% and 7.2% in the samples *Tolypothrix* sp. Prague and *Tolypothrix* sp. Tatra M [34]. Stearic acid represents the fraction of saturated fatty acids. Three of the samples contained equal amounts (2.8%), while the remaining samples had similar quantities (4.6% and 6.5%). These results are consistent with those reported by Rezanka et al. [34]: 6.3% in *Tolypothrix* sp. Prague, and 2.7% in *Tolypothrix* sp. Tatra M. The analyzed samples also contained relatively high quantities of polyunsaturated fatty acids. α-Linolenic acid was identified in low quantities (1.1–2.9%) across all analyzed species, while γ-linolenic acid was found in *Tolypothrix* sp. PACC 5501 (9.7%) and *Tolypothrix tenuis* PACC 5497 (7.7%). A similar trend was observed for stearidonic acid, which was absent only in *Tolypothrix distorta* SAG 1482-2. According to Rezanka et al. [34], the content of stearidonic acid in the analyzed *Tolypothrix* sp. samples was 2.9% and 5.4%, respectively.

**Figure 2:**
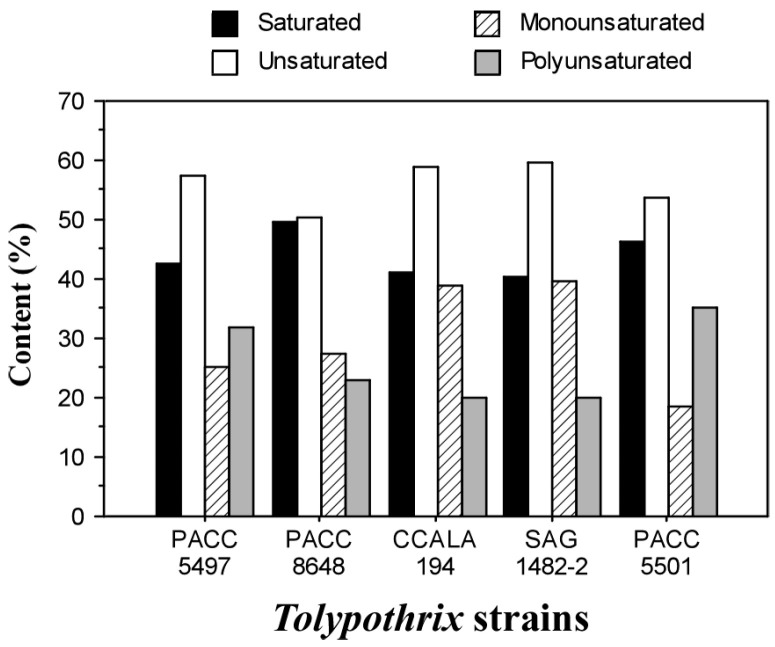
The ratio between saturated and unsaturated (mono- and poly-) acids of glyceride oil of the *Tolypothrix* strains.

The amounts of saturated (SFA), monounsaturated (MUFA), and polyunsaturated (PUFA) fatty acids were calculated. The results are presented in Figure 2.

The content of SFAs ranged from 40.4% to 49.8%, while the unsaturated fatty acids ranged from 50.2% to 59.6%. These results are close to those reported by De Oliveira et al. [35], where the SFA content was approximately 36% and the MUFA content was 50% for the cyanobacterium *Tolypothrix* sp. CACIAM 22. The total PUFA content was similar in *Tolypothrix distorta* CCALA 194, *Tolypothrix distorta* SAG 1482-2, and *Tolypothrix tenuis PACC 8648*, ranging from 19.9% to 22.8%. *Tolypothrix* sp. PACC 5501 and *Tolypothrix tenuis* PACC 5497 had PUFA contents from close to 32.0% to 35.2%.

The presence of n-3 and n-6 essential fatty acids is extremely important in the progression of disease processes [36]. The n-6/n-3 ratio was calculated for all analyzed samples (Table 2). The results showed very high values in *Tolypothrix distorta* CCALA 194 (8.14) and *Tolypothrix distorta* SAG 1482-2 (8.05), whereas *Tolypothrix tenuis* PACC 8648 stood out with the lowest ratio (0.21). The n-6/n-3 fatty acid ratio in the glyceride oil from *Tolypothrix* sp. PACC 5501 and *Tolypothrix tenuis* PACC 5497 was within the advisable range of 1:1 to 2:1 [37,38], namely 1.53 and 1.22, respectively. The data indicate that an imbalanced intake of n-6 over n-3 fatty acids could lead to chronic diseases such as diabetes, cancer, and cardiovascular diseases [39].

### 2.3. Antioxidant Activity of Tolypothrix Extracts

The antioxidant properties of non-polar extracts from all five *Tolypothrix* strains were investigated. The extracts were evaluated for their free radical scavenging capacity (2,2-Diphenyl-1-picrylhydrazyl, DPPH and 2,2′-azino-bis[3-ethylbenzothiazoline-6-sulfonic acid], ABTS), and their total phenolic content (TPC) was also assessed. The methods for testing antioxidant activity in the present study (DPPH and ABTS) are complementary. DPPH is most commonly used to assess total antiradical activity, while ABTS assesses water- and lipid-soluble (hydrophilic and lipophilic) antioxidants.

**Figure 3:**
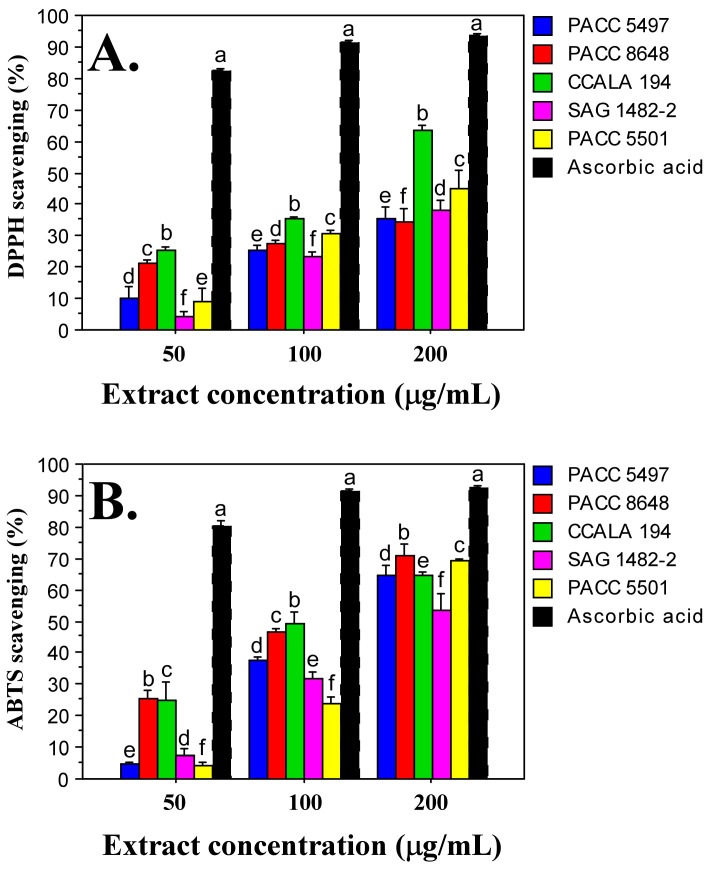
Antioxidant activity of *Tolypothrix* extracts. DPPH scavenging (**A**) and ABTS scavenging (**B**) at different concentrations (50, 100, and 200 µg/mL) of non-polar extracts from the *Tolypothrix* strains. Values are expressed as mean ± SD of three replicates. Letters show a significant difference (*p* < 0.05) according to Duncan’s test. Ascorbic acid was used as a positive control.

The DPPH radical scavenging assay is based on the use of a free radical (2,2-diphenyl-1-picrylhydrazyl), which is capable of accepting an electron or hydrogen radical and transforming into a stable molecule. In this study, the DPPH radical scavenging capacity increased with increasing extract concentration—50 µg/mL, 100 µg/mL and 200 µg/mL (Figure 3A). A dose-dependent antioxidant effect was observed for all *Tolypothrix* extracts tested. When monitoring the data at an extract concentration of 200 µg/mL, the DPPH assay showed the highest antioxidant activity in *Tolypothrix distorta* CCALA 194 strain (64%), and the lowest—in *Tolypothrix tenuis* PACC 8648 strain (35%). It should be noted that the values for *Tolypothrix tenuis* PACC 8648 (35%), *Tolypothrix tenuis* PACC 5497 (36%) and *Tolypothrix distorta* SAG 1482-2 (38%) were very close. When 100 μg/mL extract of each *Tolypothrix* strain reacts with DPPH, 35% of the initial DPPH concentration was released for *Tolypothrix distorta* strain CCALA 194, 31% for *Tolypothrix* sp. PACC 5501, 27% for *Tolypothrix tenuis* strain PACC 8648, 25% for *Tolypothrix tenuis* PACC 5497 and 23% for *Tolypothrix distorta* SAG 1482-2, respectively. A similar profile was also observed in the diagrams at an extract concentration of 50 μg/mL (Figure 3A).

The ABTS (2,2′-Azino-bis(3-ethylbenzothiazoline-6-sulfonic acid)) method evaluates the reduction of the ABTS^+^ radical cation. In this study, the antioxidant activity, similar to that reported with the DPPH method, showed a dose-dependence. The highest antioxidant activity was detected at an extract concentration of 200 µg/mL. In the strain *Tolypothrix tenuis* PACC 8648 it was 71%, followed by *Tolypothrix* sp. PACC 5501—69%. The lowest capacity for scavenging the ABTS^+^ radical cation was obtained in the strain *Tolypothrix distorta* SAG 1482-2 (53%). The strains *Tolypothrix distorta* CCALA 194 (64%) and *Tolypothrix tenuis* PACC 5497 (64%) showed identical antioxidant activity (Figure 3B). When applying the ABTS method, a similarity in the profile of the diagrams was observed at different concentrations of the tested extracts. When 100 μg/mL extract of each *Tolypothrix* strain reacted with the ABTS^+^ radical cation, 49% of the initial concentration of ABTS^+^ was released for *Tolypothrix distorta* strain CCALA 194, 46% for *Tolypothrix tenuis* strain PACC 8648, 37% for *Tolypothrix tenuis* strain PACC 5497, 32% for *Tolypothrix distorta* strain SAG 1482-2, and 24% for *Tolypothrix* sp. strain PACC 5501. A similar profile is also shown by the diagrams at an extract concentration of 50 μg/mL (Figure 3B).

The radical scavenging ability of the ABTS radical was generally higher than that of DPPH (1.4 times for strain SAG 1482-2; 1.5 times for strain PACC 5501; 1.7 times for strain PACC 5497, and 2 times for strain PACC 8648). Only in the strain *Tolypothrix distorta* CCALA 194 was the same antioxidant activity value determined by both methods—64%. In this strain, the total phenolic content was also the highest (12.502 µg/mg gallic acid equivalents, GAE).

### 2.4. Total Phenolic Content (TPC) in Non-Polar Tolypothrix Extracts

Phenolic compounds (phenolic acids, tannins and flavonoids) are major antioxidants and major components contributing to the antioxidant activity of algae. The total phenolic content (TPC) of *Tolypothrix* extracts was determined using the Folin–Ciocalteu method. This method is based on the electron transfer reaction, which allows the evaluation of the reductive antioxidant capacity of the extract. If we compare the values of the TPC in the studied extracts (Table 3), the highest TPC was recorded in the extract of strain *Tolypothrix distorta* CCALA 194 (12.49 µg/mg GAE), followed by strain *Tolypothrix distorta* SAG 1482-2 (8.79 µg/mg GAE). The lowest phenolic content was observed in the extract of strain *Tolypothrix* sp. PASS 5501 (2.10 µg/mg GAE), followed by strain *Tolypothrix tenuis* PACC 8648 (3.63 µg/mg GAE) and *Tolypothrix tenuis* PACC 5497 (4.70 µg/mg GAE). We could conclude that the representatives of the cyanobacterial species *Tolypothrix distorta* have 2–3 times higher values of TPC compared to those of the species *Tolypothrix tenuis*. The data showed that the TPC in the CHCl_3_/MeOH fractions varies. It was highest in representatives of the cyanobacterial species *Tolypothrix distorta*.

### 2.5. Anti-Inflammatory Activity of Tolypothrix Extracts

**Figure 4:**
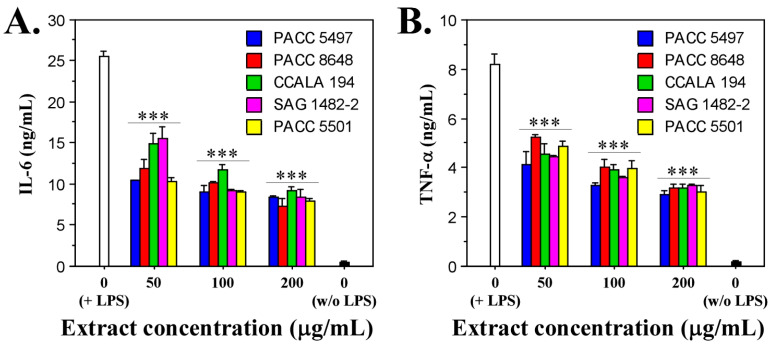
In vitro effects of *Tolypothrix* extracts on LPS-stimulated murine macrophages (RAW264.7 cells). Levels of secreted IL-6 (**A**) and TNF-α (**B**) in the cell culture medium after treatment of the LPS-stimulated cells with increasing concentrations of extracts (50, 100, and 200 µg/mL) for 24 h. The asterisks indicate statistical significance (*** *p* < 0.001) from the positive control (non-treated LPS-stimulated cells) as determined by the Kruskal–Wallis test.

To study the anti-inflammatory capacity of the non-polar extracts of the *Tolypothrix* strains, we treated lipopolysaccharides (LPS)-stimulated macrophages (RAW264.7 cell line) with a gradient concentration of the extracts (50, 100 and 200 µg/mL) for 24 h. LPS-unstimulated macrophages were used as a negative control, and LPS-stimulated macrophages not treated with extract were used as a positive control. After 24 h of exposure, the levels of pro-inflammatory cytokines IL-6 and TNF-α were measured. The data show that all the tested extracts reduced the production of both cytokines. A clear dose-dependence of the effect was observed. The data show high statistical significance (Figure 4).

The strongest inhibitory effect on IL-6 production was observed at an extract concentration of 200 µg/mL, at which for all extracts tested, the amount of cytokine secreted by macrophages ranged between 7.5 and 8 ng/mL. In comparison, the amount of IL-6 produced in the positive control was 25 ng/mL, which means a decrease of about three-fold (Figure 4A).

Similar data were obtained for the other pro-inflammatory cytokine, TNF-α. The production of this cytokine was also suppressed most strongly at the highest concentration of the extracts (200 µg/mL) and ranged between 3 and 3.5 ng/mL. These values, compared to the value of the positive control (8.2 ng/mL TNF-α), indicate a reduction in the amount of cytokine released between 2.3 and 2.7 times (Figure 4B).

The fact that the studied cyanobacterial extracts of the genus *Tolypothrix* suppress the production of both pro-inflammatory cytokines in macrophages stimulated with LPS indicates a well-pronounced anti-inflammatory effect.

### 2.6. In Vitro Cytotoxicity of Stearidonic Acid (SDA)

After establishing that non-polar *Tolypothrix* extracts have cytotoxic and anticancer effects on carcinoma cell lines (Figure 1 and Figures S1–S4), and examining the fatty acid composition of *Tolypothrix* strains (Table 2), we decided to further test the effect of stearidonic acid (SDA) on the cell lines. SDA was chosen because the analysis of the fatty acid composition of *Tolypothrix* strains showed a more interesting profile in terms of its presence and amount, varying among the strains studied. The SDA was present in all tested *Tolypothrix* strains except one—*Tolypothrix distorta* SAG 1482-2 (Table 2).

Stearidonic acid was our first choice also because it reduces the proliferation index and increases apoptosis in cancer cells, and is a precursor of eicosapentaenoic acid (EPA) and docosahexaenoic acid (DHA), which also induce apoptosis, arrest the cell cycle [40], and reduce tumor growth in vivo [41]. Our goal was to determine which component was responsible for the observed effects and, if possible, to establish the mechanism of action of the active substances contained in the extracts.

**Figure 5:**
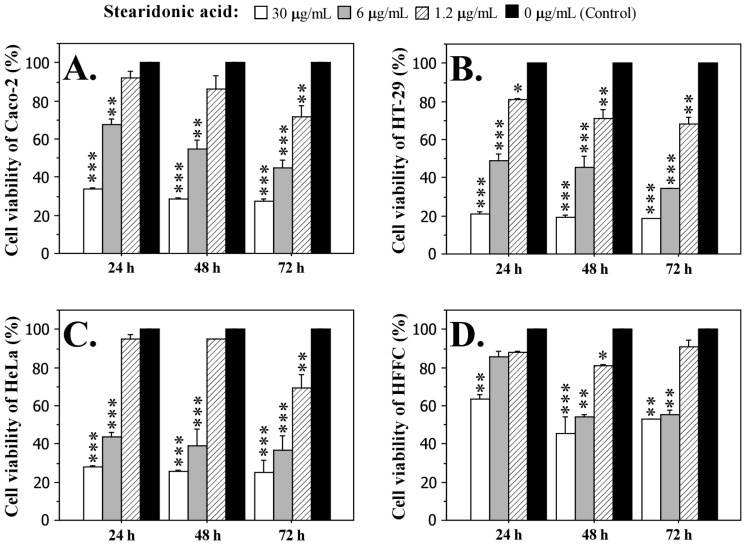
Effect of different concentrations (30 µg/mL, 6 µg/mL, and 1.2 µg/mL) of stearidonic acid (SDA) on the cell viability of adenocarcinoma Caco-2 cells (**A**), HT-29 cells (**B**), HeLa cells (**C**), and normal human fibroblasts HFFC (**D**) after treatment for 24, 48 and 72 h. Cell viability was measured by MTT assay. Data are expressed as mean ± SD. Statistically significant differences from the untreated cells (control) were determined by the Mann–Whitney *U* test. * *p* < 0.05, ** *p* < 0.01, *** *p* < 0.001.

Cells were treated with three concentrations of stearidonic acid (1.2 µg/mL, 6 µg/mL and 30 µg/mL) for 24, 48 and 72 h. The data presented in Figure 5 show clearly pronounced dose- and time-dependent cytotoxic effects, which were observed in all carcinoma cell lines and are supported by strong statistical significance. In fibroblast cells, the cytotoxic effect induced by SDA treatment was significantly weaker, indicating the specific anticancer activity of the acid. The most sensitive to the action of SDA was the HT-29 cell line, in which cell survival at the highest concentration of 30 µg/mL varied between 21.2% (for 24 h) and 18.55% (for 72 h). The IC_50_ value for this cell line was 5.18 µg/mL.

If we compare the effect of SDA on Caco-2 and HeLa cells, it is evident that the inhibitory effect is higher in HeLa cells, where the highest concentration of the fatty acid after 72 h of exposure caused a higher inhibition of vital activity and proliferation (75.2%) compared to Caco-2 cells (72.6%). The IC_50_ values for the two cell lines Caco-2 and HeLa were 10.15 µg/mL and 5.04 µg/mL, respectively. Fibroblast cells were significantly less affected by the treatment with SDA. Interestingly, the strongest toxic effect on these cells was observed at 48 h (54.4% at 30 µg/mL SDA), after which the cells seem to overcome the effect and the survival rate increased (52.7% at 30 µg/mL SDA after 72 h of treatment). The IC_50_ value of the fibroblast cell line was 17.7 µg/mL.

### 2.7. Molecular Interaction of the Stearidonic Acid (SDA)

The key mechanisms of anticancer activity involving SDA are several and they are related to molecules and receptors that appear to be targets for SDA. SDA affects inflammatory pathways by interacting with enzymes involved in lipid metabolism and with nuclear receptors. SDA inhibits the action of cyclooxogenase-2 (COX-2) and arachidonate 5-lypoxigenase (ALOX5), reducing prostaglandins (e.g., PGE2) and leukotrienes that enhance tumor growth [42,43]. In addition, SDA and its metabolites can inhibit nuclear factor kappa B (NF-kB), which reduces the expression of inflammatory cytokines (such as IL-6 and TNF-α) that contribute to tumor development [42]. SDA activates peroxisome proliferator-activated receptor gamma (PPAR-γ), acting as a ligand for the nuclear receptor involved in lipid metabolism and tumor suppression. Activation of PPAR-γ leads to cell cycle arrest and apoptosis in breast, prostate, and colon cancer cells [42,44,45].

**Figure 6:**
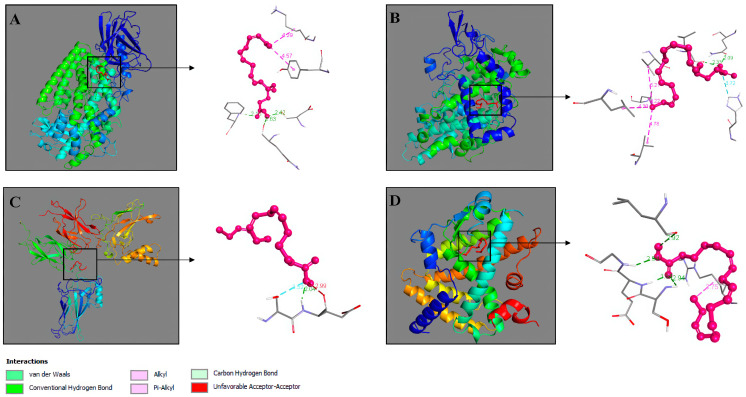
Molecular docking of SDA—ALOX5 (**A**), SDA—COX-2 (**B**), SDA—NF-κB (**C**), and SDA—PPAR-γ (**D**). Interacting complexes were visualized by Discovery Studio 2016 V16.1.0.

To further investigate the mechanism of these important interactions, a docking analysis was performed to help predict their binding. The binding of SDA to ALOX5, COX-2, NF-kB and PPAR-γ was simulated. The aim was to predict how SDA would bind to the corresponding receptors and to calculate the binding affinity. In performing the analysis, optimization of the 3D structures of the receptors and the fatty acid was performed, and the docking site was determined. Highly favored molecular interactions between the SDA and its possible targets can be seen in Figure 6.

The data from the docking analyses show that SDA binds with the highest affinity to COX-2, where a highest binding affinity energy of −7.3 kcal/mol was calculated (Table 4). The binding affinity of SDA to ALOX5 and PPAR-γ demonstrated similarity, with very close values of −6.2 kcal/mol and −6.7 kcal/mol, respectively. The docking studies of SDA with NF-κB showed only −4.2 kcal/mol (Table 4).

**Figure 7:**
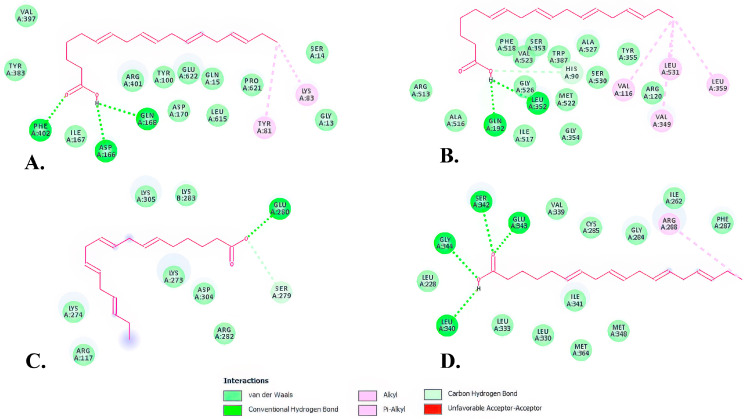
Two-dimensional (2D) structures of SDA-protein complexes representing binding affinity of SDA—ALOX5 (**A**), SDA—COX-2 (**B**), SDA—NF-κB (**C**), and SDA—PPAR-γ (**D**).

Two-dimensional structures showed that SDA formed conventional hydrogen bonds with three amino acid residues of ALOX5 (Gln168, Phe402, Asp166) (Table 4, Figure 7A, dark green) and two alkyl bonds (residues Tyr81 and Lys83) (Table 4, Figure 7A, pink).

In the SDA—COX-2 complex, there were two conventional hydrogen bonds involving Gln192 and Leu352 residues (Table 4, Figure 7B, dark green) and four hydrophobic (alkyl) bonds involving Val116, Val349, Leu359 and Leu531 residues (Table 4, Figure 7B, pink). One carbon hydrogen bond, involving the His90 amino acid residue was also formed between the SDA and COX-2 complex (Table 4, Figure 7B, light green). All of these bonds result in SDA having the highest affinity the COX-2 enzyme.

The SDA—PPAR-γ interactions were formed by four conventional hydrogen bonds and one alkyl bond. The residues involved in hydrogen bonds were Leu340, Ser342, Glu343 and Gly344 (Table 4, Figure 7D, dark green), while the alkyl bond involved an Arg288 residue.

The SDA—NF-κB interaction showed the lowest binding affinity among the interactions. SDA and NF-κB formed a conventional hydrogen bond at Glu280 and a carbon hydrogen bond at Ser279 (Table 4, Figure 7C, dark green and light green, respectively). Interestingly, the docking simulation showed an unfavorable acceptor–acceptor interaction at Glu280, which perhaps plays an important role in the regulation of this interaction.

## 3. Discussion

### 3.1. Cytotoxicity and Anticancer Potential of Non-Polar Extracts from Tolypothrix Strains

The cytotoxic effects of non-polar extracts of cyanobacteria on human cells in vitro are the subject of active scientific research. These extracts, obtained by organic solvents such as methanol, chloroform, dichloromethane and hexane, contain various biologically active compounds, including higher fatty acids, which can exhibit cytotoxicity and induce oxidative stress in cell lines. In the present study, treatment of three carcinomas and one normal cell line with non-polar extracts of five *Tolypothrix* strains resulted in dose- and time-dependent cytotoxic effects in all cancer cell lines after exposure for 24, 48 and 72 h at different concentrations (50, 100 and 200 µg/mL) (Figure 1).

The most pronounced inhibitory effect on cell viability was observed in the HT-29 cell line, with an IC_50_ value of 106.27 µg/mL (Figure 1B). We believe that the cyanobacterial extracts have selective cytotoxicity, since when normal cells were exposed to the same treatment conditions as the tumor cell lines, they did not show a significant decrease in cell viability, which means that the cyanobacterial extracts are not toxic to normal cells. Such effects after treatment with extracts from the genus *Tolypothrix* are presented here for the first time. Similar experiments, but with extracts from *Aphanothece halophytica*, were conducted by Silva et al. [46]. The extracts (mixture of ethyl acetate: methanol (1:1)) were tested in vitro for cytotoxicity on four human cancer cell lines—T98G (glioblastoma), MDA231 (breast cancer), A549 (lung cancer), K562 (leukemia)—and normal human fibroblasts (BJ-5ta). The extracts showed significant cytotoxic activity towards the MDA231 cancer cells, but not towards the normal cell line [46]. In the same extracts of *Aphanothece halophytica*, higher fatty acids were identified, including hexadecanoic acid (C_16:0_), hexadecenoic acid (C_16:1_) and stearic acid (C_18:0_), which are major components of the non-polar fractions. These compounds could interact with cell membranes and disrupt their integrity, leading to cell death. We also found the same and many other higher fatty acids in the studied *Tolypothrix* extracts (Table 2).

Another group of scientists showed that non-polar extracts of marine cyanobacteria, such as *Synechocystis* and *Synechococcus*, obtained with organic solvents (methanol and dichloromethane), induce apoptosis in HL-60 (human monocytic leukemia cells) [47]. The cytotoxic effect was more pronounced with the dichloromethane extract compared to the methanol extract. When treated with some of the extracts, up to 90% of the cells were apoptotic. Our previous studies on the biological activity of extracts of the cyanobacterium *Fischerella major* showed that the most sensitive to exposure to *Fischerella* extracts were HeLa cells, where the toxicity of Extract 3 (chloroform/methanol) at a concentration of 100 µg/mL after 24 h exposure reached 70%. After 48 and 72 h of treatment of the cells with the same extract and concentration, the toxicity increased to 86% and 92%, respectively. Extract 3 also showed the lowest IC_50_ value—26.8 µg/mL—after a 72 h exposure of HeLa cells [48]. It is clear that different extracts exhibit different degrees of cytotoxicity, which is probably due to the presence of different biologically active compounds with different polarity and nature. Approximately 30% of cyanobacterial extracts have been reported to cause damage to mammalian cells in vitro [49]. This damage may be caused by the presence of specific secondary metabolites or synergistic effects of compounds that affect cellular metabolism [46].

The chemical composition of cyanobacterial extracts includes different classes of compounds, such as fatty acids, alkaloids, hydrocarbons and oxygenated compounds. Higher fatty acids, such as hexadecanoic acid (C_16:0_), hexadecenoic acid (C_16:1_) and stearic acid (C_18:0_), quite well represented in all *Tolypothrix* extracts, are among the main components of the non-polar fractions and can interact with cell membranes, leading to cell death. These fatty acids were identified for the first time in the *Tolypothrix* strains studied.

### 3.2. Antioxidant Activity

Antioxidant activity in photosynthetic organisms (including cyanobacteria) is part of their strategy for protecting against the action of free radicals and oxidative stress. Given their nature—photosynthetic autotrophs—these organisms are good candidates for antioxidant producers. Cyanobacteria and microalgae are rich in bioactive compounds. Studying their antioxidant potential is important due to the applications they could have. Cyanobacteria are considered potential sources of natural antioxidants that are suitable for applications in the food, pharmaceutical and cosmetic industries. This also explains our interest in the cyanobacterial genus *Tolypothrix* as a source of antioxidant substances, an aspect of this genus which is still poorly studied. The methods for studying the antioxidant activity of *Tolypothrix* strains in the present study are DPPH and ABTS, supplemented by TPC measurement.

In the present study, non-polar extracts of five *Tolypothrix* strains were tested. *Tolypothrix distorta* CCALA 194 showed the highest DPPH radical scavenging capacity (64%) at an extract concentration of 200 µg/mL, and the highest total phenolic content (12.49 µg/mg GAE).

Studies have shown that chloroform and chloroform/methanol extracts of *Anabaena flos-aquae* exhibit significant antioxidant activity. For example, the chloroform extract showed DPPH activity of 8.63 ± 0.71 mg Trolox Equivalent (TE)/g, while the chloroform/methanol extract showed 4.82 ± 0.34 mg TE/g, respectively. The reported total phenolic content in the two extracts was 29.57 ± 1.11 and 18.21 ± 0.31 mg GAE/g, respectively [50]. DPPH analysis of a CHCl_3_/MeOH extract of a *Fischerella major* strain demonstrated 25% DPPH activity [48], and a total phenolic content of 100 µg/mL GAE. In general, similar data for non-polar cyanobacterial extracts are scarce, due to the preference of researchers for working with polar extracts. The present study enriches the information related to the antioxidant activity of non-polar extracts.

Furthermore, studies on marine cyanobacteria have shown that chloroform and acetone extracts have high antioxidant activity, with acetone extracts showing higher levels of phenolic compounds than ethyl acetate extracts [51]. The values cited above, as well as those reported in the present study, are far from the TPC value of 43 mg/g GAE found in the cyanobacterial species *Spirulina platensis*, which has been shown to be a source of antioxidants and is widely used in various industries [52]. However, it should be taken into account that antioxidant activity is not determined solely by phenols.

Unlike higher plants, which lack metabolic pathways for the production of polyunsaturated fatty acids (PUFAs) with 20 or more C-atoms, bacteria (including cyanobacteria), microalgae and fungi have the ability to synthesize C20, C22n-3 and C22n-6 PUFAs, such as eicosapentaenoic acid (EPA), arachidonic acid (AA), docosapentaenoic acid (DPA) and docosahexaenoic acid (DHA) [44].

Higher fatty acids are generally not well known for their antioxidant activity, as most of them are susceptible to oxidation. However, some unsaturated fatty acids and their metabolites may exert indirect antioxidant activity, mainly by modulating cell signaling, expression of antioxidant enzymes, and interaction with receptors. Higher fatty acids with reported antioxidant activity are the ω-3 unsaturated fatty acids—alpha-linolenic acid (ALA), docosahexaenoic acid (DHA), eicosapentaenoic acid (EPA), and the ω-9 unsaturated oleic acid [53,54,55,56]. Alpha-linolenic acid (ALA), identified in the present study in three *Tolypothrix* strains (PACC 5497, CCALA 194, and PACC 5501), could have indirect antioxidant activity by regulating gene expression, reducing inflammation and oxidative stress. Docosahexaenoic acid (DHA), found in strain PACC 5501, has been associated with a reduction in oxidative stress in cells by increasing the expression of antioxidant enzymes such as glutathione peroxidase [57]. The monounsaturated ω-9 oleic acid, found in all *Tolypothrix* strains studied and in quite high amounts (between 10.2% in *Tolypothrix* sp. PACC 5501 and 21.8% in strain *Tolypothrix distorta* CCALA 194), has anti-inflammatory and antioxidant properties, mainly through the regulation of the cell membrane and signaling pathways [56].

Non-polar cyanobacterial extracts exhibit not only antioxidant activity but also other biological effects. Studies have shown that these extracts may have anti-inflammatory, antimicrobial and cytoprotective properties [58,59]. For example, extracts from *Anabaena flos-aquae* show cytoprotective effects against oxidative stress, making them potential candidates for biotechnological applications [60].

### 3.3. Anti-Inflammatory Activity

Non-polar cyanobacterial extracts have shown anti-inflammatory effects on the macrophage cell line RAW264.7, a commonly used in vitro model for studying inflammatory processes [31,32,61]. Publications related to the biological activity of cyanobacteria often point to these organisms as a source of substances with anti-inflammatory activity [62]. Representatives of the genus *Tolypothrix* also exhibit such activity. The species *Tolypothrix nodosa*, for example, produces the diterpenoid tolypodiol, which has anti-inflammatory activity in the mouse ear edema assay [30].

In the present study, a clearly expressed dose-dependent anti-inflammatory effect of the *Tolypothrix* strains was observed. It was assessed on the basis of the suppressed production of the pro-inflammatory cytokines IL-6 and TNF-α in LPS-stimulated macrophages (cell line RAW264.7). Similar to the effect obtained by us, Ku et al. [32] reported a decrease in the expression of IL-6 and TNF-α in LPS-stimulated macrophages (RAW264.7) after treatment with 50 µg/mL lipid extract of the cyanobacteria *Nostoc commune* and *Spirulina platensis*. The same authors also reported the ability of both extracts to reduce the translocation of NF-kB in macrophages.

Non-polar compounds, such as 7(E)-9-keto-octadec-7-enoic acid (C18 acid), isolated from cyanobacteria, suppressed iNOS expression and nitric oxide (NO) production in RAW264.7 cells stimulated with LPS. This suggests inhibition of the NF-κB signaling pathway. At the same time, increased expression of Nqo1 was observed, associated with activation of the antioxidant pathway Nrf2/ARE, which contributes to the anti-inflammatory effect [20].

TNF-α plays an important role in the disruption of macrovascular and microvascular circulation, both in vivo and in vitro, and is an important cytokine that can induce apoptosis and inflammatory processes [63]. On the other hand, NF-kB belongs to a family of inducible transcription factors that regulate a large set of genes involved in various processes of immune and inflammatory responses [63,64].

The anti-inflammatory effects of non-polar cyanobacterial extracts are associated with: (1) inhibition of the NF-κB pathway through suppression of iNOS expression and reduced NO production, which leads to the limitation of the inflammatory response; and (2) activation of the Nrf2/ARE pathway through increased expression of antioxidant genes, such as Nqo1, which contributes to cellular protection against oxidative stress and inflammation [20]. Given the data we obtained (suppression of and reduction in IL-6 and TNF-α production), we can assume that the anti-inflammatory activity in this case is a result of inhibition of the NF-κB signaling pathway. However, this does not exclude the second alternative, given the reported pronounced antioxidant activity of the studied extracts. It is clear that future additional studies are needed to prove this hypothesis.

In conclusion, we can say that non-polar cyanobacterial extracts demonstrate significant anti-inflammatory potential in the macrophage cell line RAW264.7 by modulating key inflammatory and antioxidant pathways. These data highlight the possibility of developing novel natural anti-inflammatory agents based on cyanobacterial metabolites, including those from *Tolypothrix* strains.

### 3.4. In Vitro Effects of Stearidonic Acid (SDA)

The anticancer activity demonstrated in the treatment of the cell lines Caco-2, HT-29 and HeLa with a graded concentration of SDA (1. 2 µg/mL, 6 µg/mL and 30 µg/mL) was not surprising. Polyunsaturated fatty acids from the ω-3 group, of which stearidonic acid (SDA) is a representative, have been found to have anti-carcinogenic effects, in contrast to that of ω-6 PUFAs, whose effects are pro-carcinogenic [65,66]. Publications show that ω-3 PUFAs, and in particular SDA, suppress the proliferation of cancer cells and stimulate apoptosis [42,67]. Changes in membrane fluidity affect tumor progression and metastasis.

While saturated fatty acids (SFA) reduce membrane permeability, monounsaturated fatty acids (MUFA) and polyunsaturated fatty acids (PUFA) promote membrane fluidity. It is suggested that SDA exerts its anticancer effect through multiple mechanisms—modulation of inflammation, induction of apoptosis, modulation of lipid metabolism, changes in membrane lipid composition, and activation of tumor suppressor receptors [44].

Since the effect of stearidonic acid in our experiment was assessed with MTT, a method that allows us to assess mitochondrial integrity, we assume that, in this case, SDA affects the mitochondrial pathway of apoptosis activation (intrinsic apoptosis). Stearidonic acid can increase the expression of pro-apoptotic proteins (BAX), while anti-apoptotic proteins (Bcl-2) decrease, leading to mitochondrial membrane permeabilization and cytochrome-c release. This activates caspase-3 and caspase-9, triggering apoptosis in tumor cells [42,43].

Summarizing the results of molecular docking, we could speculate that SDA exerts its antitumor effects as follows: (1) reducing pro-inflammatory eicosanoids (by inhibiting COX-2 and ALOX5); (2) activating PPAR-γ, leading to metabolic stress and cell cycle arrest/interruption; (3) altering the properties of cancer cell membranes (disruption of lipid rafts); and (4) inducing apoptosis (by BAX, Bcl-2 and caspases). These data suggest that SDA could regulate the signaling pathways mediated by the studied target enzymes or the transcription factor NF-κB.

## 4. Materials and Methods

### 4.1. Cyanobacterial Cultivation and Preparation of Extracts

Five *Tolypothrix* strains (Nostocales, Cyanobacteria) were used in the present study—*Tolypothrix tenuis* PACC 5497, *Tolypothrix tenuis* PACC 8648, *Tolypothrix distorta* CCALA 194, *Tolypothrix distorta* SAG 1482-2, and *Tolypothrix* sp. PACC 5501. They were obtained from the Plovdiv Algal Culture Collection (PACC), established and maintained at Paisii Hilendarski University of Plovdiv, Bulgaria. All strains were provided in the form of freeze-dried biomass. The cyanobacteria were seeded in 75 cm^2^ culture flasks (TPP, Trasadingen, Switzerland) in alkaline Z-medium [68] and cultured under sterile conditions. A photoperiod of 12 h light/12 h darkness was maintained during the cultivation process. Light with 10 μmol s^−1^m^−2^ photon intensity was generated using 40 W cool-white fluorescent lamps.

To obtain cyanobacterial biomass the expanded cultures were collected and transferred into centrifuge tubes. After centrifugation for 15 min at 4000 rpm, the cell pellet was frozen and freeze-dried. A total of 500 mg of freeze-dried cyanobacterial biomass from each *Tolypothrix* strain was used to prepare the extracts. The material was mixed with 3 mL methanol and vortexed for 1 min. This was followed by extraction in an ultrasonic bath (Branson 5510R-DTH, Wilmington, NC, USA) for 20 min under periodical vortexing. Then, 6 mL of chloroform was added to and mixed with the resulting suspension, which was then shaken for 20 min at 15 rpm. After that, 3 mL of Milli-Q water was added and the mixture was vortexed for 1 min. The extracts were centrifuged for 20 min at 4000 rpm. Then, the methanol/chloroform (non-polar) and water/methanol (polar) fractions were separated and filtered through a 0.20 µm hydrophobic PTFE filter Millex-FG (Merck KGaA, Darmstadt, Germany). Organic solvents were removed by evaporation to dryness under a vacuum at 37 °C (Savant SpeedVac Concentrator, SAVANT Instruments Inc., Farmingdale, NY, USA). The dried extracts were dissolved in DMSO/water solution (1:1) to a final concentration of 5 mg/mL (*w*/*v*). To achieve a final concentration of DMSO < 1%, working solutions for the in vitro assays were prepared with a Dulbecco’s modified Eagle’s medium (DMEM) or Dulbecco’s Phosphate-Buffered Saline (DPBS) purchased from Gibco^®^, Life Technologies™, Paisley, Scotland, UK.

### 4.2. Cell Lines

The cytotoxic and antitumor potential of the obtained extracts was assessed in vitro using four human cell lines—Caco-2 (human colorectal adenocarcinoma, ATCC HTB-37™), HT-29 (human colorectal adenocarcinoma, ATCC HTB-38™), HeLa (human cervical adenocarcinoma, ATCC CCL-2™), and HFFC (human foreskin fibroblast cells, provided by CLS Cell Lines Service GmbH, Eppelheim, Germany). A mouse macrophage cell line, RAW 264.7 (ATCC TIB-71™), was used to evaluate the anti-inflammatory activity of the extracts. All cells were grown in Dulbecco’s modified Eagle’s medium (DMEM) containing 10% heat-inactivated fetal bovine serum (FBS) and antibiotics (100 U/mL penicillin and 100 µg/mL streptomycin) (all provided by Merck KGaA, Darmstadt, Germany). Cell cultures were grown and manipulated under sterile conditions. The cells were initially expanded in 75 cm^2^ flasks and cultured at 37 °C using incubators with 5% carbon dioxide supply and high humidity.

### 4.3. In Vitro Cytotoxicity Assays

To perform in vitro cytotoxicity assays, 5 × 10^4^ cells/mL suspensions from the four cell lines were prepared and 200 µL/well were seeded on 96-well plates (TPP, Trasadingen, Switzerland). After 24 h, the *Tolypothrix* extracts or stearidonic acid (SDA) were added to the cells. The treatment was carried out in triplicate using different concentrations of the extracts (50, 100, and 200 µg/mL) or SDA (30 µg/mL, 6 µg/mL, and 1.2 µg/mL). An equivalent amount of DMSO/water solution (1:1) was added to the control wells in each test plate. Cells were treated for 24, 48 or 72 h. Then, the cytotoxic effect of the extracts was measured by the MTT (3-(4,5-dimethylthiazol-2-yl)-2,5-diphenyl tetrazolium bromide) test. MTT and SDA were purchased from Merck KGaA, Darmstadt, Germany.

### 4.4. MTT Test

At the end of the treatment with extract samples (24, 48 or 72 h), 20 μL of 5 mg/mL MTT solution was added to the cells. The plates were incubated at 37 °C for 2 h in darkness. After that, the culture medium was aspirated and 100 μL of dimethyl sulfoxide (DMSO) was added to each well in order to dissolve the formazan product generated in the cells during incubation with MTT. The plates were briefly placed on a shaker for 10–15 min, and after that, the absorbance at 570 nm was measured using the SpectraMax i3x instrument (Molecular devices, San Jose, CA, USA). Percent viability and inhibition of cell metabolic activity were calculated using the detected absorbance units from each test sample and the control cells grown for the time period in standard culture medium. Extract concentrations that induced 50% cellular inhibition (IC_50_) were determined.

### 4.5. Determination of Lipid Content and Fatty Acid Composition

The lipids were extracted from the samples with a mixture of chloroform and methanol (2:1, *v*/*v*) applying the method described by Breil et al. [69].

The fatty acid composition of fats was determined by gas chromatography (GC) [70]. Fatty acid methyl esters (FAMEs) were prepared by pre-esterification of the samples with 2% sulfuric acid in absolute methanol at 50° [71]. Determination of FAMEs was performed on an Agilent 8860 gas chromatograph (Agilent, Santa Clara, CA, USA) equipped with a capillary column DB FastFAME (30 m × 0.25 mm × 0.25 µm (film thickness)) (Agilent, Santa Clara, CA, USA) and a flame ionization detector. The column temperature was raised from 70 °C (1 min) to 180 °C at 6 °C/min speed, and at 5 °C/min to 250 °C; the temperature of the injector was 270 °C and that of the detector was 300 °C; nitrogen was used as the carrier gas. Identification was carried out by comparison of the retention times of a standard mixture of FAMEs. The fatty acid composition was presented as a percentage of total fatty acids.

### 4.6. Antioxidant Activity (DPPH and ABTS Assays)

The antioxidant activity of the cyanobacterial extracts was evaluated based on their ability to scavenge two different radicals—DPPH (2,2-diphenyl-1-picrylhydrazyl) radical and ABTS (3-ethylbenzothiazoline-6-sulphonic acid) diammonium salt) cation radical.

The DPPH assay was conducted based on the method described by Ichikawa et al. [72]. First, 100 μL of extract was mixed with 100 μL 50% ethanol solution of 2 mM DPPH. The samples were incubated in the dark for 30 min. Then, the reduction in DPPH absorption was measured at 520 nm using a SpectraMax i3x spectrophotometer (Molecular devices, San Jose, CA, USA). Ascorbic acid solution in different concentrations served as a positive control. All samples were assayed in triplicate. The DPPH radical scavenging activity was calculated by the following formula:

% DPPH scavenging = [(Ac − As)/Ac)] × 100

where Ac denotes the absorbance of the control (DPPH solution only), and As is the absorbance of the test sample mixed with DPPH solution.

For the ABTS radical scavenging activity experiments, 7 mM ABTS (F. Hoffmann-La Roche Ltd, Basel, Switzerland) stock solution was mixed 1/1 (*v*/*v*) with 2.45 mM potassium persulfate (Merck KGaA, Darmstadt, Germany). The mixture was incubated for 4 to 16 h at 4 °C in darkness until the reaction of ABTS cation radical (ABTS^•+^) generation was complete, which was verified by measurement of absorbance at 730 nm (0.70 ± 0.05). Then, 100 µL of extract sample was mixed with 900 µL ABTS^•+^ solution. After 10 min, absorbance was read at 730 nm using a SpectraMax i3x spectrophotometer (Molecular devices, San Jose, CA, USA). Ascorbic acid was used as a positive control. All samples were examined in triplicate. The percent ABTS radical scavenging activity was determined using the following formula:

% ABTS scavenging = [(Ac − As)/Ac) × 100

where Ac denotes the absorbance of the control (ABTS^•+^ solution only), and As is the absorbance of the test sample mixed with ABTS^•+^ solution.

### 4.7. Total Phenolic Content (TPC)

The total phenolic content of *Tolypothrix* extracts was determined using the Folin–Ciocalteu method as described by Batsalova et al. [73] with slight modifications. Briefly, *Tolypothrix* extracts were pipetted into a 96-well plate at a gradient concentration of 25, 50 and 100 µg/mL. Then, 200 µL of 2% Na_2_CO_3_ solution and 50 µL of Folin–Ciocalteu solution were added to each well. Incubation was then carried out in the dark for 2 h at room temperature. The absorbance of the formed blue complex was measured at 765 nm with a spectrophotometer (SpectraMax i3x, Molecular devices, San Jose, CA, USA). Gallic acid in serial dilutions (500 µg/mL, 250 µg/mL, 125 µg/mL, 62.5 µg/mL, 31.25 µg/mL, 15.625 µg/mL, 7.8 µg/mL) was used to obtain a standard curve. Total phenolic content was calculated according to the standard curve and expressed as µg/mg gallic acid equivalents (GAE). All samples were pooled and analyzed in triplicate.

### 4.8. Anti-Inflammatory Activity

Experiments with a mouse macrophage cell line were performed to assess the anti-inflammatory activity of the *Tolypothrix* extracts. RAW 264.7 cells were seeded on a 96-well culture plate (TPP, Trasadingen, Switzerland) and cultured overnight. Then, the cells were treated with *Tolypothrix* extracts and 1 µg/mL LPS for 24 h. In addition, a positive control was assayed—cells cultured for the same period in medium containing only LPS to induce inflammatory activity. The secretion of pro-inflammatory cytokines in the cell culture medium was assessed by enzyme-linked immunosorbent assay (ELISA). For this purpose, LEGEND MAX™ Mouse IL-6 and TNF-α ELISA kits (BioLegend, San Diego, CA, USA) were used, and the assays were performed according to the protocol provided by the manufacturer. The concentration of cytokines secreted by RAW 264.7 cells in the culture medium was calculated using standard curves.

### 4.9. Molecular Interaction Analysis

The Molecular docking with Autodock Vina (via PyRx 0.8) [74] was used to evaluate interactions between SDA and other molecules/receptors: stearidonic acid with arachidonate 5-lipoxygenase (ALOX5), cyclooxygenase-2 (COX-2), nuclear factor kappa B (NF-κB), and peroxisome proliferator-activated receptor gamma (PPAR-γ). The three-dimensional structure of the stearidonic acid (PubChem CID:5312508) was downloaded from the PubChem (https://pubchem.ncbi.nlm.nih.gov/, accessed on 16 March 2025) in SDF format [75]. The crystal structures of ALOX5 (PDB ID: 3o8y), COX-2 (PDB ID: 5kir), NF-κB (PDB ID: 3do7) and PPARγ (PDB ID: 3et3) were retrieved from the RCSB protein data bank (https://www.rcsb.org/, accessed on 16 March 2025) in PDB format [76]. Discovery Studio 2016 V16.1.0 (BIOVIA, Dassault Systèmes, San Diego, CA, USA) was used to separate complex structures, followed by removal of water molecules, heteroatoms, hydrogens, and Gasteiger–Marsili charges [77]. UCSF Chimera 1.14 performed energy minimization for the structures, and OpenBabel converted files into pdbqt format for docking [78,79]. Default docking parameters were applied, and the best-docked complex was selected based on the lowest energy score (kcal/mol). Interactions were analyzed and visualized with Discovery Studio Visualizer [80] (BIOVIA, Dassault Systèmes, San Diego, CA, USA) and PyMOL [81] (SourceForge, San Diego, CA, USA).

### 4.10. Statistics

The obtained results were presented as mean values ± standard deviation (SD) or standard error of the mean (SEM) and compared to those from the controls. For this purpose, the Mann–Whitney U-test or Kruskal–Wallis test was applied using the StatView software (version 5.0; SAS Institute Inc., Cary, NC, USA). Data from the DPPH and ABTS assays were analyzed with one-way analysis of variance (ANOVA) followed by Duncan’s post hoc test (IBM SPSS Statistics, version 28.0. Armonk, NY, USA). Values of *p* lower than 0.05 were considered significant.

## 5. Research Limitations and Future Directions

Despite the growing number of studies related to the biological activity of components isolated from cyanobacteria, there are still limitations that should not be overlooked. First of all, it should be taken into account that different strains of the same cyanobacterial species may contain different biologically active substances (including fatty acids) and in different amounts. Therefore, it is necessary to find the most suitable strains that contain or produce sufficient quantities of the desired bioactive component. The accurate identification of bioactive components is a challenge for researchers working with natural products, since many of these components are unique and there are no reference standards to compare them with. This makes such studies very expensive, requiring long procedures and analyses. For this reason, many of the studies were performed at the extract level, without identifying the active components. Identifying the structures of bioactive components would also facilitate the study of mechanisms of action. Therefore, future research directions in this area should be related to the development of standardized methods for extraction and analysis of bioactive components from cyanobacteria, which would ensure repeatability and comparability of the results. If it is impossible to synthesize the active components due to their complex nature, at least it should be possible to isolate and purify them in sufficient quantities for industrial application.

## 6. Conclusions

Microalgae and cyanobacteria are still an insufficiently well-studied source of natural products. The data on the strains of the cyanobacterial genus *Tolypothrix* presented in this study are a good basis for future research and possible biotechnological applications. The results obtained by us clearly and categorically demonstrate the presence of significant anticancer, antioxidant and anti-inflammatory effects of the studied non-polar extracts, which is new information. For the first time, the exact qualitative and quantitative composition of the fatty acids of the tested non-polar extracts for these strains has been determined. The data demonstrate in a categorical manner the anticancer activity of stearidonic acid, which is part of the fatty acid composition of the extracts. Molecular docking showed the possible interaction between SDA and its target molecules and receptors (ALOX5, COX-2, NF-kB and PPAR-γ). The search for natural sources of biologically active substances is extremely important and topical. The creation of medicines and dietary supplements on this basis has a number of advantages such as harmlessness, synergy and economic efficiency. Therefore, we believe that the results presented by us demonstrate scientific value and significance by enriching knowledge on this subject and enabling future practical applications.

## Figures and Tables

**Table 1:** Glyceride oil content of five samples of *Tolypothrix* strains.

Sample	*Tolypothrix tenuis* PACC 5497	*Tolypothrix tenuis* PACC 8648	*Tolypothrix distorta* CCALA 194	*Tolypothrix distorta* SAG 1482-2	*Tolypothrix* sp. PACC 5501
Oil content (% in the wet sample)	0.72 ± 0.22	0.97 ± 0.31	0.31 ± 0.14	0.45 ± 0.25	1.09 ± 0.37

**Table 2:** Individual fatty acid composition of glyceride oil of five samples of *Tolypothrix* strains.

Fatty Acids (%)		*Tolypothrix tenuis* PACC 5497	*Tolypothrix tenuis* PACC 8648	*Tolypothrix distorta* CCALA 194	*Tolypothrix distorta* SAG 1482-2	*Tolypothrix* sp. PACC 5501
C_8:0_	Caprylic	1.1 ± 0.1	1.5 ± 0.3	4.2 ± 0.2	5.1 ± 0.1	0.3 ± 0.0
C_10:0_	Capric	0.5 ± 0.2	0.7 ± 0.1	2.8 ± 0.3	2.8 ± 0.2	0.2 ± 0.0
C_11:0_	Undecanoic	1.7 ± 0.4	0.6 ± 0.1	0.8 ± 0.2	0.3 ± 0.0	-
C_12:0_	Lauric	0.3 ± 0.1	0.9 ± 0.2	0.5 ± 0.1	0.3 ± 0.0	1.3 ± 0.2
C_13:0_	Tridecanoic	0.2 ± 0.0	0.3 ± 0.0	0.4 ± 0.0	0.6 ± 0.1	0.3 ± 0.0
C_14:0_	Myristic	1.1 ± 0.2	3.5 ± 0.3	0.7 ± 0.1	0.9 ± 0.2	2.8 ± 0.3
C_14:1_	Myristoleic	0.3 ± 0.1	0.4 ± 0.1	0.3 ± 0.0	0.3 ± 0.0	0.2 ± 0.0
C_15:0_	Pentadecanoic	0.2 ± 0.0	1.1 ± 0.2	0.5 ± 0.1	0.6 ± 0.2	1.5 ± 0.2
C_15:1_	Pentadecenic	0.5 ± 0.2	0.4 ± 0.0	0.2 ± 0.0	0.3 ± 0.0	0.4 ± 0.1
C_16:0_	Palmitic	32.0 ± 0.5	35.2 ± 0.3	21.3 ± 0.5	24.9 ± 0.5	36.2 ± 0.4
C_16:1_	Palmitoleic	2.3 ± 0.3	9.2 ± 0.2	13.5 ± 0.3	15.9 ± 0.4	6.3 ± 0.3
C_16:2_ n-6	7,10-hexadienoic	0.4 ± 0.1	0.6 ± 0.1	1.4 ± 0.1	1.6 ± 0.2	0.4 ± 0.1
C_17:0_	Margaric	0.7 ± 0.2	0.5 ± 0.1	1.8 ± 0.2	2.1 ± 0.1	0.7 ± 0.2
C_16:3_ n-3	7,10,13-Hexadecatrienoic	0.8 ± 0.2	0.9 ± 0.2	0.8 ± 0.1	0.9 ± 0.2	0.9 ± 0.1
C_17:1_	Heptadecenoic	1.2 ± 0.1	1.1 ± 0.1	3.0 ± 0.3	3.6 ± 0.2	1.3 ± 0.2
C_18:0_	Stearic	4.6 ± 0.3	2.8 ± 0.1	6.5 ± 0.2	2.8 ± 0.1	2.8 ± 0.2
C_18:1_	Oleic	21.0 ± 0.5	16.3 ± 0.3	21.8 ± 0.5	19.6 ± 0.4	10.2 ± 0.3
C_18:2_ n-6	Linoleic	7.7 ± 0.2	18.2 ± 0.2	16.1 ± 0.3	16.1 ± 0.3	11.2 ± 0.4
C_18:3_ n-6	γ-Linolenic	7.7 ± 0.3	-	0.4 ± 0.1	-	9.7 ± 0.2
C_18:3_ n-3	α-Linolenic	2.9 ± 0.4	2.9 ± 0.3	1.1 ± 0.1	1.3 ± 0.2	2.3 ± 0.1
**C_18:4_ n-3**	**Stearidonic**	**10.7 ± 0.5**	**0.2 ± 0.0**	**0.3 ± 0.0**	**-**	**8.5 ± 0.3**
C_20:0_	Arachidic	-	-	1.3 ± 0.2	-	-
C_20:2_ n-6	Eicosadienoic	1.8 ± 0.3	-	-	-	-
C_22:0_	Behenic	0.3 ± 0.0	2.3 ± 0.2	0.3 ± 0.0	-	0.3 ± 0.1
C_22:6_ n-3	Docosahexaenoic	-	-	-	-	2.2 ± 0.2
C_24:0_	Tetracosanoic	0.4 ± 0.1	-	-	-	-
Omega-6 (n-6)		17.6	4.0	17.9	17.7	21.3
Omega-3 (n-3)		14.4	18.8	2.2	2.2	13.9
Ratio n-6/n-3		1.22	0.21	8.14	8.05	1.53

**Table 3:** Total phenolic content in non-polar extracts of five samples of *Tolypothrix* strains.

Sample	*Tolypothrix tenuis* PACC 5497	*Tolypothrix tenuis* PACC 8648	*Tolypothrix distorta* CCALA 194	*Tolypothrix distorta* SAG 1482-2	*Tolypothrix* sp. PACC 5501
µg/mg GAE	4.70 ± 0.91	3.63 ± 0.33	12.49 ± 0.16	8.79 ± 0.64	2.10 ± 0.13

**Table 4:** Binding affinity and interacting bonds of stearidonic acid (SDA) to ALOX5, COX-2, NF-κB and PPAR-γ.

Targets	Binding Affinity	Type of Interacting Bonds	Distance (Å)
ALOX5	−6.2 kcal/mol	Conventional hydrogen bonds	
		N:UNK1:H-A:GLN168:O *	2.63
		A:PHE402:HN-N:UNK1:O	2.15
		N:UNK1:H-A:ASP166:O	2.42
		Alkyl bonds	
		A:TYR81:HH-N:UNK1:C	4.29
		A:LYS83:HH-N:UNK1:C	5.57
COX-2	−7.3 kcal/mol	Conventional hydrogen bonds	
		N:UNK1:H-A:GLN192:OE1	2.09
		N:UNK1:H-A:LEU352:O	2.33
		Alkyl bonds	
		N:UNK1:C-A:VAL116	4.22
		N:UNK1:C-A:VAL349	4.99
		N:UNK1:C-A:LEU359	4.34
		N:UNK1:C-A:LEU531	3.91
		Carbon hydrogen bonds	
		A:HIS90:CE1-N:UNK1:O	3.72
NF-κB	−4.2 kcal/mol	Conventional hydrogen bonds	
		A:GLU280:HN-N:UNK1:O	2.04
		Carbon hydrogen bonds	
		A:SER279:CB-N:UNK1:O	3.52
PPAR-γ	−6.7 kcal/mol	Conventional hydrogen bonds	
		N:UNK1:H-A:LEU340:O	2.91
		A:SER342:HN-N:UNK1:O	2.94
		A:GLU343:HN-N:UNK1:O	1.83
		A:GLY344:HN-N:UNK1:O	2.54
		Alkyl bonds	
		A:ARG288-N:UNK1	3.75

* UNK1 (unknown ligand) = stearidonic acid (SDA).

## Data Availability

The original contributions presented in this study are included in the article/Supplementary Material.

## Supplementary Materials

The following supporting information can be downloaded at: https://www.mdpi.com/article/10.3390/ijms26115086/s1.

## Abbreviations

The following abbreviations are used in this manuscript:

PACC	Plovdiv Algal Culture Collection
CCALA	Culture Collection of Autotrophic Organisms
SAG	Sammlung von Algenkulturen (Culture Collection of Algae)
MTT	3-(4,5-dimethylthiazol-2-yl)-2,5-diphenyl tetrazolium bromide
DPPH	2,2-Diphenyl-1-picrylhydrazyl
ABTS	2,2′-Azino-bis(3-ethylbenzothiazoline-6-sulfonic acid)
LPS	Lipopolysaccharides
IL-1β	Interleukin-1 beta
IL-6	Interleukin 6
TNF-α	Tumor necrosis factor alpha
IC_50_	Half-maximal inhibitory concentration
DMSO	Dimethyl sulfoxide
DPBS	Dulbecco’s Phosphate-Buffered Saline
TPC	Total phenolic content
GAE	Gallic acid equivalents
SFAs	Saturated fatty acids
MUFAs	Monounsaturated fatty acids
PUFAs	Polyunsaturated fatty acids
SDA	Stearidonic acid
EPA	Eicosapentaenoic acid
DHA	Docosahexaenoic acid
ALA	Alpha-linolenic acid
AA	Arachidonic acid
DPA	Docosapentaenoic acid
NO	Nitric oxide
NF-κB	Nuclear factor kappa B
COX-2	Cyclooxygenase-2
ALOX5	Arachidonate 5-lipoxygenase
PPAR-γ	Peroxisome proliferator-activated receptor gamma
