# Supplementary material for: Tolypothrix Strains (Cyanobacteria) as a Source of Bioactive Compounds with Anticancer, Antioxidant and Anti-Inflammatory Activity
Source: Int J Mol Sci. 2025 May 26;26(11):5086. doi: 10.3390/ijms26115086 (PMC12154164; doi:10.3390/ijms26115086)
Supplement: Supplementary file 1 [file ijms-26-05086-s001.zip › ijms-3659182-supplementary.pdf]

Article

Tolypothrix Strains (Cyanobacteria) as a Source of Bioactive Compounds with Anticancer, Antioxidant and Anti-Inflammatory Activity

Ivanka Teneva [1], Tsvetelina Batsalova [2], Dzhemal Moten [2], Zhana Petkova [3], Olga Teneva [3], Maria Angelova-Romova [3], Ginka Antova [3] and Balik Dzhambazov [2,*]

[1] Department of Botany and Biological Education, Faculty of Biology, Paisii Hilendarski University of Plovdiv, 24 Tsar Assen Str., 4000 Plovdiv, Bulgaria; teneva@uni-plovdiv.bg (I.T.)
[2] Department of Developmental Biology, Faculty of Biology, Paisii Hilendarski University of Plovdiv, 24 Tsar Assen Str., 4000 Plovdiv, Bulgaria; tsvetelina@uni-plovdiv.bg (T.B.); moten@uni-plovdiv.bg (D.M.)
[3] Department of Chemical Technology, Faculty of Chemistry, Paisii Hilendarski University of Plovdiv, 24 Tsar Assen Str., 4000 Plovdiv, Bulgaria; zhanapetkova@uni-plovdiv.bg (Z.P.); olga@uni-plovdiv.bg (O.T.); maioan@uni-plovdiv.bg (M.A-R.); ginant@uni-plovdiv.bg (G.A.)
* Correspondence: balik@uni-plovdiv.bg; Tel.: +359-32-262-535

Abstract: Cyanobacterial extracts offer significant potential for the development of new natural antioxidants and biologically active compounds with applications in various industries. Data on the genus *Tolypothrix* are limited, therefore the aim of the present study was to investigate the anticancer, antioxidant and anti-inflammatory activity of extracts prepared from strains of this genus. Cytotoxicity and anticancer activity were evaluated by in vitro tests with four cell lines using the MTT assay. The assessment of antioxidant activity was performed by the DPPH and ABTS methods in combination with the calculation of the total phenolic content. Anti-inflammatory activity was investigated using the LPS-stimulated macrophage model (RAW264.7) and subsequent measurement of the levels of secreted cytokines IL-6 and TNF-α. The lipid content and fatty acid composition of the non-polar extracts were determined by gas chromatography (GC). To elucidate the mechanism of cytotoxicity/anticancer action of the non-polar extracts, the effects of stearidonic acid, which was detected in four of the studied cyanobacterial strains, were additionally tested on the same cell lines. A molecular docking analysis was performed simulating the interaction between stearidonic acid and its target molecules and receptors (ALOX5, COX-2, NF-kB and PPAR-γ). In all cancer cell lines (but not in the normal one), dose-dependent cytotoxic effects were observed after exposure to different concentrations of non-polar *Tolypothrix* extracts. The most pronounced inhibitory effect was observed on the HT-29 cell line, with an IC_{50} value of 106.27 μg/mL. A dose-dependent antioxidant effect was established for all tested extracts, measured by both DPPH and ABTS methods. All non-polar extracts reduced the production of pro-inflammatory cytokines IL-6 and TNF-α in LPS-stimulated macrophages RAW264.7 and the effect was dose-dependent. Analysis of the fatty acid composition revealed 26 different fatty acids. Our conclusion is that the *Tolypothrix* strains exhibit anticancer, antioxidant, and anti-inflammatory activity and they could be a promising source for the production of natural products.

Keywords: *Tolypothrix*; cyanobacteria; fatty acids; cytotoxicity; anticancer activity; antioxidant activity; anti-inflammatory activity

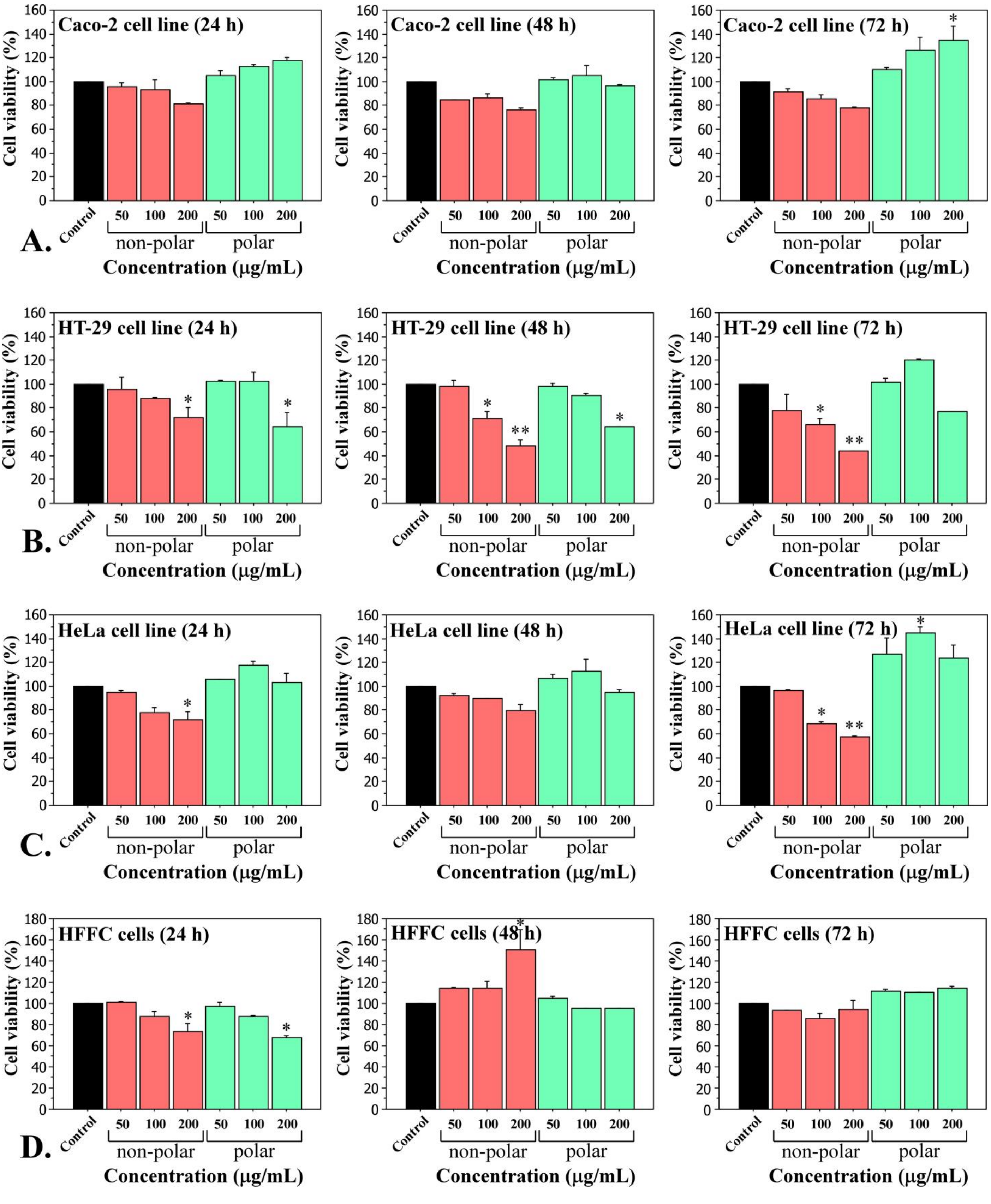

Figure S1. Cytotoxicity of *Tolypothrix tenuis* PACC 8648 extracts on adenocarcinoma Caco-2 cells (**A**), HT-29 cells (**B**), HeLa cells (**C**) and normal human fibroblasts HFFC (**D**). Cell viability was measured using MTT assays after treatment with increasing concentrations of extracts (50, 100, and 200 μg/mL) for 24, 48 and 72 h. Results are expressed as mean ± SD of three independent experiments, each performed in triplicate. Statistical significance was defined by the Mann–Whitney U test versus the control. * $p < 0.05$, ** $p < 0.01$.

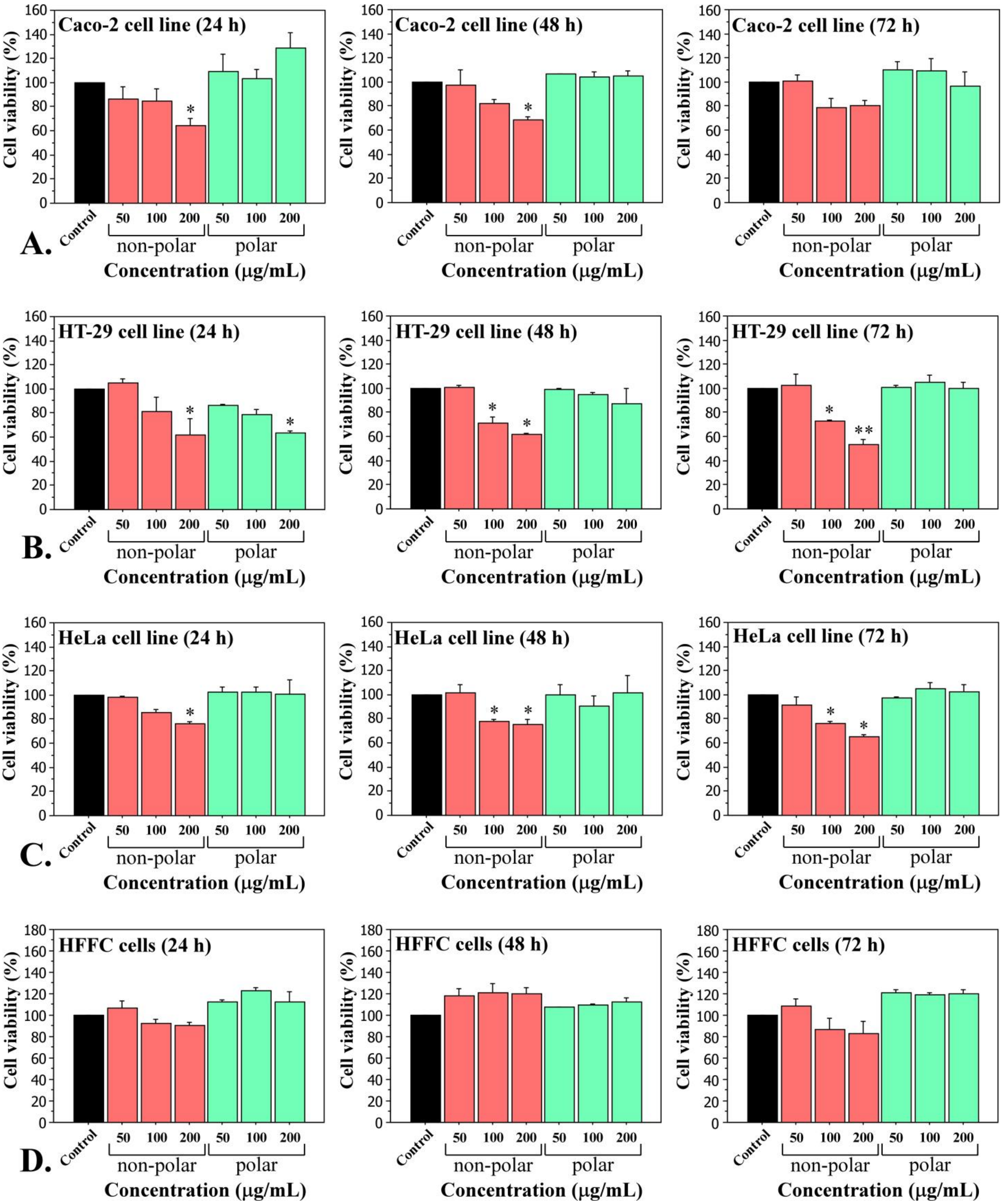

Figure S2. Cytotoxicity of *Tolypothrix distorta* CCALA 194 extracts on adenocarcinoma Caco-2 cells (**A**), HT-29 cells (**B**), HeLa cells (**C**) and normal human fibroblasts HFFC (**D**). Cell viability was measured using MTT assays after treatment with increasing concentrations of extracts (50, 100, and 200 μg/mL) for 24, 48 and 72 h. Results are expressed as mean ± SD of three independent experiments, each performed in triplicate. Statistical significance was defined by the Mann–Whitney U test versus the control. * $p < 0.05$, ** $p < 0.01$.

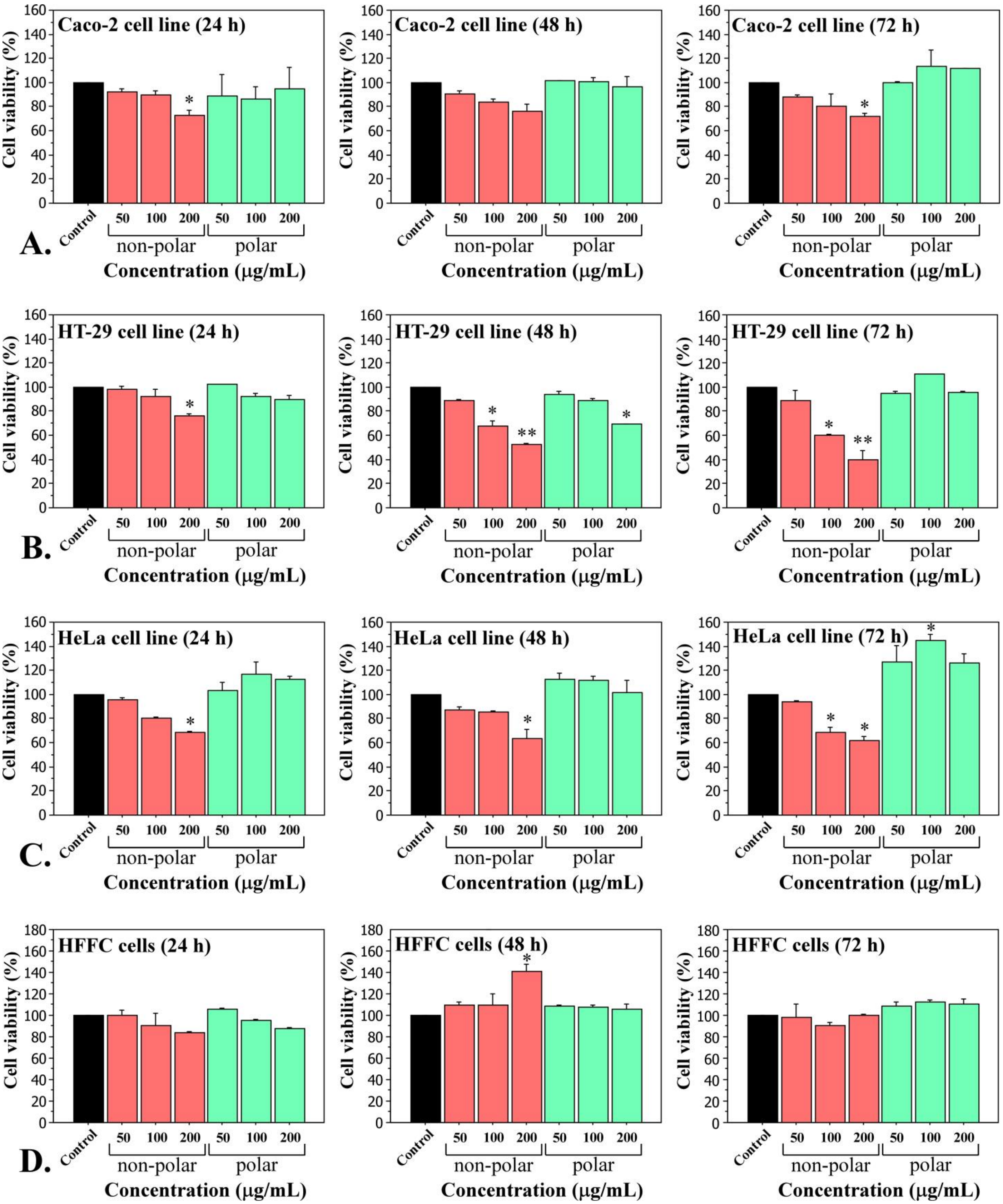

Figure S3. Cytotoxicity of *Tolypothrix distorta* SAG 1482-2 extracts on adenocarcinoma Caco-2 cells (**A**), HT-29 cells (**B**), HeLa cells (**C**) and normal human fibroblasts HFFC (**D**). Cell viability was measured using MTT assays after treatment with increasing concentrations of extracts (50, 100, and 200 μg/mL) for 24, 48 and 72 h. Results are expressed as mean ± SD of three independent experiments, each performed in triplicate. Statistical significance was defined by the Mann–Whitney U test versus the control. * $p < 0.05$, ** $p < 0.01$.

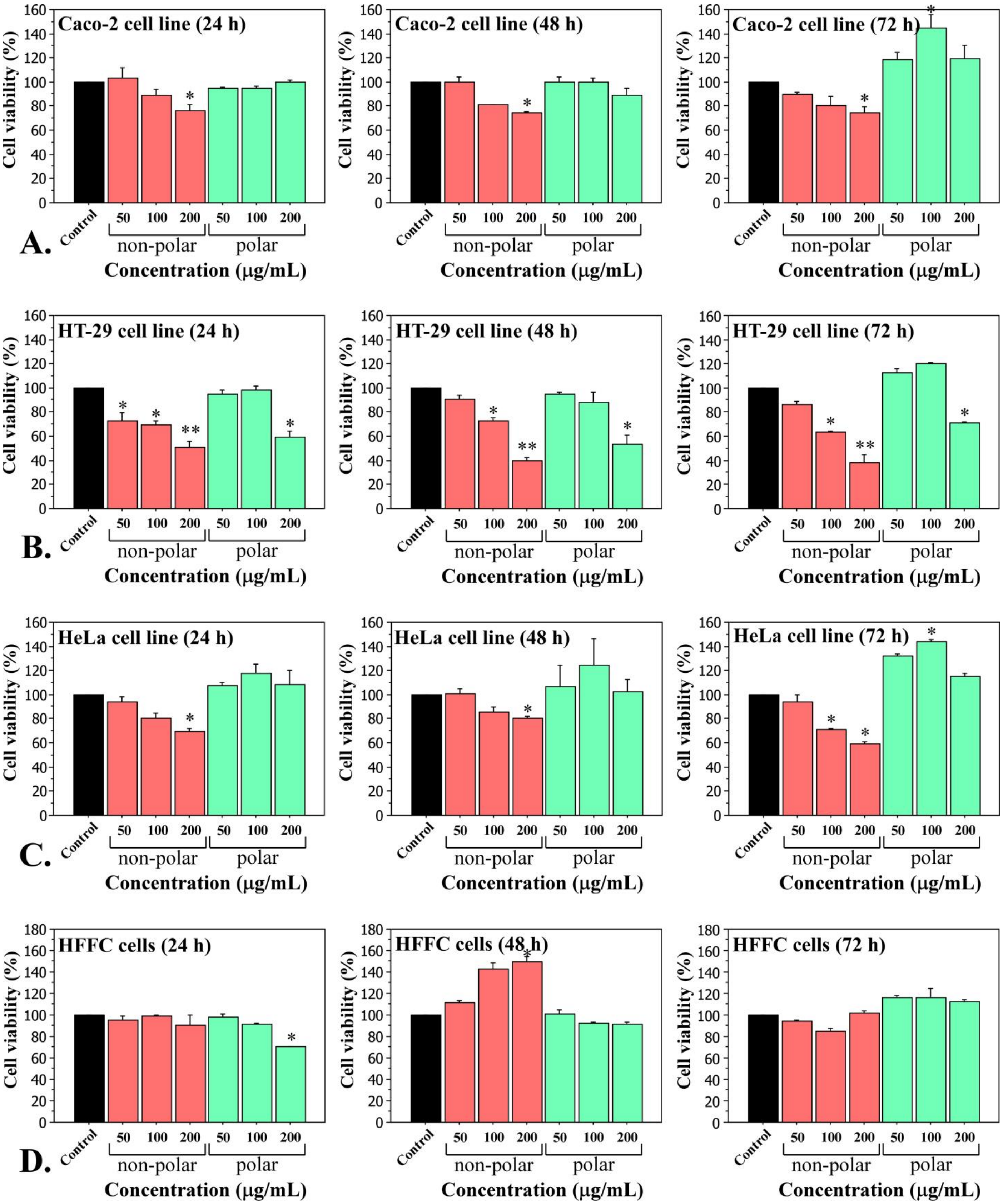

Figure S4. Cytotoxicity of *Tolypothrix sp.* PACC 5501 extracts on adenocarcinoma Caco-2 cells (**A**), HT-29 cells (**B**), HeLa cells (**C**) and normal human fibroblasts HFFC (**D**). Cell viability was measured using MTT assays after treatment with increasing concentrations of extracts (50, 100, and 200 μg/mL) for 24, 48 and 72 h. Results are expressed as mean ± SD of three independent experiments, each performed in triplicate. Statistical significance was defined by the Mann–Whitney U test versus the control. * $p < 0.05$, ** $p < 0.01$.
